# Functional Validation of an Alpha-Actinin-4 Mutation as a Potential Cause of an Aggressive Presentation of Adolescent Focal Segmental Glomerulosclerosis: Implications for Genetic Testing

**DOI:** 10.1371/journal.pone.0167467

**Published:** 2016-12-15

**Authors:** Di Feng, Julia M. Steinke, Ramaswamy Krishnan, Gabriel Birrane, Martin R. Pollak

**Affiliations:** 1 Division of Nephrology, Department of Medicine, Beth Israel Deaconess Medical Center and Harvard Medical School, Boston, Massachusetts, United States of America; 2 Division of Pediatric Nephrology and Transplantation, Helen DeVos Children's Hospital at Spectrum Health, Grand Rapids, Michigan, United States of America; 3 Department of Emergency Medicine, Beth Israel Deaconess Medical Center and Harvard Medical School, Boston, Massachusetts, United States of America; 4 Division of Experimental Medicine, Department of Medicine, Beth Israel Deaconess Medical Center and Harvard Medical School, Boston, Massachusetts, United States of America; University of Houston, UNITED STATES

## Abstract

Genetic testing in the clinic and research lab is becoming more routinely used to identify rare genetic variants. However, attributing these rare variants as the cause of disease in an individual patient remains challenging. Here, we report a patient who presented with nephrotic syndrome and focal segmental glomerulosclerosis (FSGS) with collapsing features at age 14. Despite treatment, her kidney disease progressed to end-stage within a year of diagnosis. Through genetic testing, an Y265H variant with unknown clinical significance in alpha-actinin-4 gene (ACTN4) was identified. This variant has not been seen previously in FSGS patients nor is it present in genetic databases. Her clinical presentation is different from previous descriptions of ACTN4 mediated FSGS, which is characterized by sub-nephrotic proteinuria and slow progression to end stage kidney disease. We performed in vitro and cellular assays to characterize this novel ACTN4 variant before attributing causation. We found that ACTN4 with either Y265H or K255E (a known disease-causing mutation) increased the actin bundling activity of ACTN4 in vitro, was associated with the formation of intracellular aggregates, and increased podocyte contractile force. Despite the absence of a familial pattern of inheritance, these similar biological changes caused by the Y265H and K255E amino acid substitutions suggest that this new variant is potentially the cause of FSGS in this patient. Our studies highlight that functional validation in complement with genetic testing may be required to confirm the etiology of rare disease, especially in the setting of unusual clinical presentations.

## Introduction

Alpha-actinin-4 (ACTN4) is a cytoskeleton protein that crosslinks actin filaments, helps anchor the actin cytoskeleton to focal adhesions, and provides structural support for cells[1]. Mutations in the actin-binding domain of ACTN4 cause an autosomal dominant form of focal segmental glomerulosclerosis (FSGS)[2, 3]. Several independent mutations in ACTN4 have been found to segregate with disease in FSGS families. These mutations are located within the actin-binding domain of the ACTN4, and include K255E, T259I, S262P, W59R and I149del variants. A germline mosaicism ACTN4 S262F mutation was reported in the father of two affected siblings[4]. FSGS and podocyte foot enfacement are observed in a Actn4 K256E knock-in mouse model (a mutation analogous to the FSGS-causing K255E mutation in humans), supporting the causal role of this ACTN4 mutation in human FSGS[5].

In this report, we describe a patient who presented with the full features of the nephrotic syndrome at age 14 years. A kidney biopsy demonstrated the histologic diagnosis of FSGS with collapsing features. By means of routine genetic screening for disease-causing mutations in known FSGS genes, a heterozygous ACTN4 Y265H mutation was identified. This mutation is located at a known ACTN4 phosphorylation site. Rare variants of unclear clinical significance are common in all human genes. In the absence of other available affected family members in whom to test segregation of the variant with disease, we examined this Y265H variant experimentally. Functional readouts support a causal relationship between ACTN4 Y265H mutation and FSGS in this patient. The patient’s rapid progression to end stage renal disease (ESRD) highlights the inadequacy of current treatments to treat FSGS and slow the progression of FSGS to ESRD, particularly when FSGS occurs as a result of a cytoskeleton abnormality.

## Materials and Methods

### Human Genetic Studies

Participants were enrolled in these studies after providing written informed consent for participation in our protocol, which has been approved by the Institutional Review Board at Beth Israel Deaconess Medical Center, Boston. DNA was extracted from saliva. Standard Sanger sequencing of PCR amplified genomic DNA was used to confirm the ACTN4 mutation.

### F-Actin Bundling Assays

The bundling assay was performed using the Actin Binding Protein Spin-Down Assay Biochem Kit (Cytoskeleton BK013) according to the manufacturer’s protocol. Specifically, 1.6 μM of full length ACTN4 protein was mixed with 9 μM of filamentous-actin (F-actin), incubated at room temperature for 30 minutes, and then centrifuged at 14,000g for 1 hour at 24°C. Proteins in the supernatants and pellets after centrifugation were solubilized in equal amounts of SDS sample buffer, boiled, and then subjected to 4–20% SDS-PAGE gel (Biorad). The abundance of F-actin in the pellet and supernatant was quantified by ImageJ.

### Traction Force Microscopy (TFM) Measurements

Immortalized human podocyte cells were cultured in RPMI medium (Thermo Fisher Scientific) supplemented with 10% FBS, Antibiotic-Antimycotic Solution (Corning), and ITS Liquid Media Supplement (Sigma) [6]. Contractile forces generated by podocytes were quantified by the traction force microscopy, as described previously[7]. All experiments were carried out under permissive conditions. Briefly, human podocyte podocytes were transfected with ACTN4-EGFP plasmid constructs harboring the variants of interest. After 24 hours of transfection, the cells were seeded on collagen-coated elastic polyacrylamide (PAA) substrates with stiffness 26 kilopascal (kPa). The substrates were prepared with surface-bound 0.1 μm sulfate-modified polystyrene fluorescent beads (Sigma). Podocytes were allowed to adhere for 5 h before the traction force experiments. Podocyte contractility induced the displacement of beads underneath the cells. Using an inverted fluorescent microscope (Leica DMI 6000), fluorescent images of beads were first taken when the cells were attached and then after the cells were removed. The change in the position of the beads from when the cells were attached and when they were removed was used to compute a displacement field. From the displacement field, an *a priori* knowledge of substrate stiffness, and a manual trace of the cell boundary, contractile forces were computed using the approach known as constrained traction cytometery[7]. From the contractile force map, we computed the average cellular contractile force as the root mean squared value.

### Immunofluorescence Staining

24 h after transfection, cells were trypsinized and seeded onto the 26 kPa PAA subtrates. Podocytes were allowed to adhere for 5 h before being fixed in 4% paraformaldehyde, permeabilized in 0.1% Triton X-100, and blocked with BlockAid^™^ Blocking Solution (Thermo Fisher Scientific). Podocytes were stained with rhodamine phalloidin (Thermo Fisher Scientific). Nuclei were stained with Hoechst 33342 (Thermo Fisher Scientific). Confocal images were acquired using a Zeiss LSM 880 confocal system equipped with a plan-Apochromat 63x/1.40 oil objective lens.

## Results

A 14-year-old European American girl with a 3-year history of hypertriglyceridemia was undergoing a routine yearly visit. She complained of intermittent ankle and facial swelling. Upon further evaluation, she was found to have slightly elevated blood pressure (130/82), elevated serum creatinine of 2.17 mg/dL (normal 0.4–0.9 mg/dL), leading to nephrology consultation. Further testing showed hypoalbuminemia (1.5 g/dL; normal range 3.5–5.0 g/dL) and nephrotic range proteinuria (urine protein/creatinine 11.69 g/m^2^/day; normal <0.20 g/m^2^/day). She did not have significant hematuria or cellular casts in her urine. Serum complement levels were normal. She was found to have a horseshoe kidney by ultrasound, some hydronephrosis, but no evidence of urinary obstruction on mercaptoacetyltriglycine renogram and no vesicoureteral reflux on voiding cystourethrogram.

**Fig 1:**
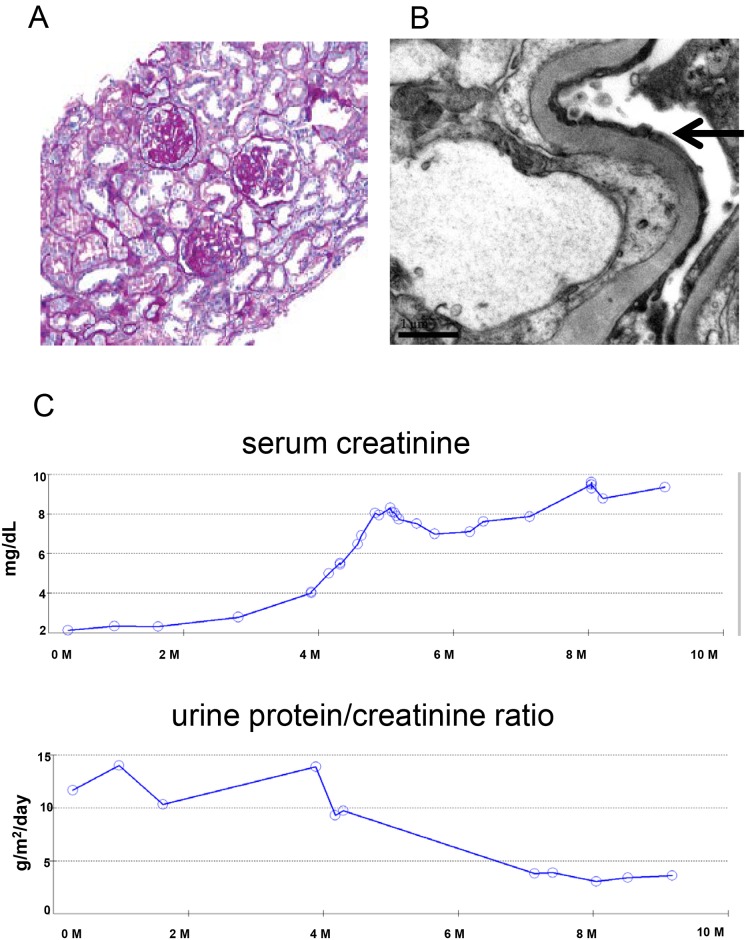
(A), histologic findings in the patient’s kidney biopsy. Periodic acid–Schiff (PAS) staining (magnification 200X) shows segmental glomerulosclerosis and interstitial fibrosis with multiple foci of microcystic tubular dilation. (B), Electron microscopy images shows glomerular podocyte foot processes effacement (arrows) (magnification 20000X). (C), serum creatinine and urine protein/creatinine ratio of the patient over a period of a year.

A kidney biopsy was performed. Histological exam demonstrated FSGS with collapsing features (Fig 1A). Electron microgram showed diffuse podocyte effacement (Fig 1B). The patient was initiated on therapy with prednisone. She was resistant to steroid treatment, creatinine increased (Fig 1C) and after six weeks, the prednisone was tapered. In addition to prednisone, she was treated with an angiotensin-converting enzyme inhibitor (enalapril). In the ensuing months, treatment also included an angiotensin II receptor blocker (losartan), a beta blocker (metoprolol), and a calcium channel blocker (amlodipine) for blood pressure control. Her kidney function deteriorated rapidly. Within 6 months of initial nephrology evaluation, her serum creatinine level had risen to 9 mg/dL. Peritoneal dialysis was started. She underwent bilateral nephrectomy at age of 15. One year later at age 16, she received a kidney transplant from a deceased donor. Two years after transplantation, the allograft is functioning without any sign of disease recurrence. Biopsy of the transplanted allograft has been performed twice since transplantation without any sign of recurrent FSGS.

**Fig 2:**
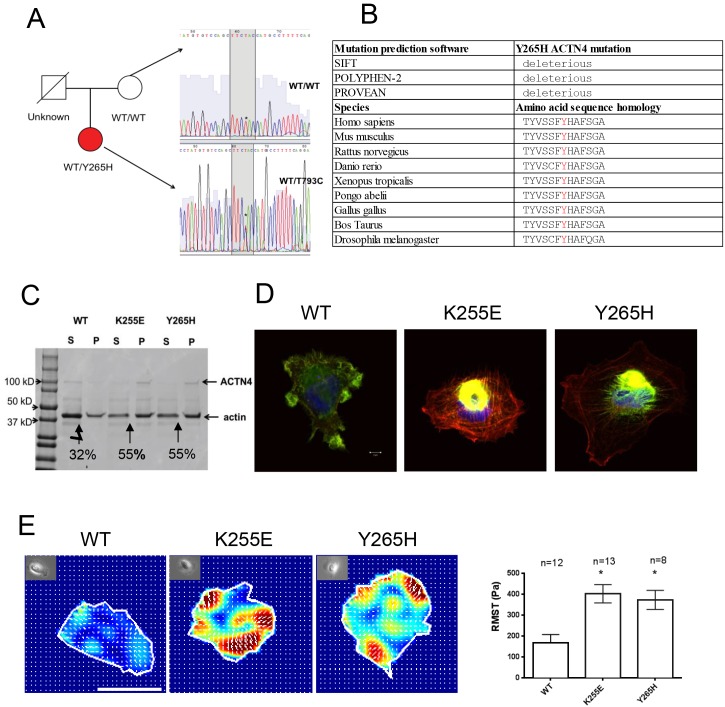
(A), pedigree of the patient and nuclear family. Partial ACTN4 genomic sequence from the patient (red circle) shows a heterozygous T793C substitution that results in a Y265H ACTN4 amino acid substitution. The patient’s mother (white circle) does not carry this mutation. The genotype of the father (white square) is unknown. (B), mutation prediction software results for Y265H ACTN4 mutation and sequence alignment showing sequence conservation of ACTN4 Y265 across difference species. (C), representative image of coomassie blue stained SDS-PAGE gel showing an F-actin bundling experiment. The distribution of ACTN4 and F-actin in supernatant (S) and pellet (P) after centrifugation is shown. Arrows point out the bundled F-actin in the pellet. 55% of F-actin (42kDa) is bundled when crosslinked by K255E ACTN4 and Y265H ACTN4 proteins while only 32% of F-actin is bundled when crosslinked by WT ACTN4. (D), representative immunofluorescence staining images. Rhodamine phalloidin stains actin (red). GFP tagged ACTN4 is green. Hoechst 33342 stains the nuclei (blue). K255E ACTN4 and Y265H ACTN4 form aggregates within the cells. Scale bar is 5μm shown in WT merged image. 2E, representative contractile force maps for podocyte expressing the different forms of ACTN4. Different colors correlate with different levels of force, with red being the largest degree of force. Arrows are force vectors. Bright field image of each cell is included in top left of each force map. Root mean square value (RMST, an index of contractile force) is plotted. Data are from one of the three independent experiments, and are expressed as mean ± SEM. *P<0.05 indicate significant difference from WT.

In an effort to identify the cause of FSGS in this patient, mutational analysis of ACTN4, INF2, TRPC6, and NPHS2 was performed by a commercial genetic testing laboratory. The result revealed a heterozygous Y265H variant in ACTN4, which is not previously reported as a causal gene of FSGS. The patient’s mother lacked this mutation. Her father passed away at age of 39 of sudden cardiac death. Like the patient, he had a history of hypertriglyceridemia and hypercholesterolemia. His DNA was not available for testing. We confirmed this result by Sanger sequence (Fig 2A). Provean, Polyphen, and SIFT prediction software predicts this amino acid change as deleterious [2, 8, 9](Fig 2B). Additionally, this variant is very rare as it is not listed in either the 1000 Genomes database, nor the Exome Aggregation Consortium database (ExAC)[10].

We next performed several in vitro and cellular assays to help us assess the likelihood that this variant was in fact causally related to the patient’s glomerulopathy. To examine the functional significance of the Y265H substitution, we first performed an in vitro F-actin bundling assay. We have previously reported that disease-causing mutant forms of ACTN4 increase the binding affinity of the actin-binding domain of ACTN4 to F-actin[2, 3]. By means of its actin binding activity, ACTN4 is able to crosslink and bundle actin filaments. We found more bundled F-actin when F-actin was incubated with either Y265H ACTN4 or K255E ACTN4 compared to the WT ACTN4 (Fig 2B). To visualize the effect of ACTN4 on actin in podocytes, we transfected human podocytes with plasmids encoding different forms of ACTN4. We found that Y265H ACTN4 behaves similarly to the disease-causing mutant K255E. Like the K255E variant, Y265H forms cytosolic ACTN4-actin aggregates within the cells (Fig 2). Additionally, we observed a clear increase in the amount of stress fibers present in the mutant ACTN4 podocytes. Finally, to determine if Y265H ACTN4 mutant affects cellular mechanics, we used traction force microscopy to measure the contractile force generated by individual podocytes. We observed that podocytes, transfected with either K255E (386.9 ± 43.54 Pa, mean ± SEM) or Y265H (366.8V± 32.98 Pa) ACTN4 were more contractile than podocytes transfected with WT ACTN4 (151.7 ± 20.22 Pa) (Fig 2D). There is no difference in cell-spread area.

## Discussion

In this report, we described a patient with an aggressive form of FSGS with a Y265H mutation in ACTN4. Ideally, a complete assessment of whether an ACTN4 mutation is disease-causing would include the following: 1) Does the mutant form abnormal cellular aggregates in the cell? 2) Does the mutant increase the binding affinity of ACTN4 to F-actin? 3) Does the mutant occur in an evolutionarily conserved domain of ACTN4? 4) Does the mutation co-segregate with the affected individuals of the family in which it was identified? In the current case, Y265H ACTN4 meets the first three criteria. However, no other affected family member was available for evaluation. We performed additional cellular assays to determine if the Y265H ACTN4 mutation changes the biophysical properties of the podocyte similarly as the previous known disease-causing mutant K255E ACTN4. The known disease-causing ACTN4 mutation K255E leads to a more contractile fibroblast [11]. Because FSGS is a disease of podocyte dysfunction, we tested the effect of both the K255E and Y265H mutations on podocyte contractility[1]. We found that both the K255E and Y265H mutations lead to more contractile podocytes when compared to the wild-type ACTN4. If in fact these cell culture findings correlate with in vivo biology, increased podocyte contractility could lead to altered glomerular function and disease.

Previous reports suggest that most of ACTN4-mediated FSGS has a late adult onset and is slowly progressive to ESRD [2, 3]. In contrast, this report describing a 14-year-old girl with FSGS presents a case of ACTN4-mediated FSGS that is of early onset with rapid progression to ESRD. Moreover, the severity of proteinuria and hypoalbuminemia at an early stage distinguishes it from previously known disease-causing ACTN4 mutations. These observations highlight an unusual clinical presentation of ACTN4-mediated FSGS. This mutation, along with two other mutations (W59R and S262F ACTN4) reported in the literature, suggests that not all ACTN4-mediated FSGS follows a similar onset and time course [3, 4]. However, it is certainly possible that the horseshoe kidney made her more susceptible to FSGS than with an ACTN4 mutation alone, and may explain the particularly aggressive nature of the disease. It is less likely that the horseshoe kidney was the primary cause of her FSGS. Horseshoe kidneys can lead to vesicoureteral reflux, a known association with FSGS, but reflux was not observed in this patient[12, 13]. An independent association between horseshoe kidneys and FSGS has not yet been found, as there have only been a few case reports presenting these two conditions together[14, 15].

Despite the increasing belief in the medical community that new technologies such as exome and genome sequencing will solve a large fraction of medical mysteries, differentiating between a rare variant unrelated to disease and a disease-causing mutation is difficult. This case raises two important issues vis-a-vis genetic testing and the need to perform functional assessments of novel variants. Firstly, clinical genetic testing laboratories are in general reluctant to call a rare variant “disease-causing”, even if it is rare and in a known disease-causing gene, unless that specific variant has been reported to be disease-causing in the published literature. However, in the case of rare Mendelian diseases, particularly dominant diseases, most disease-causing mutations are rare. Thus the absence of published information in the literature is not evidence against causation. Secondly, as with any clinical test, the prior probability of a result influences its interpretation. In the current case, the prior probability that this patient is an ACTN4-mediated form of FSGS is low because of the atypical clinical presentation and overall low prevalence of ACTN4-mediated FSGS. Therefore, merely finding the Y265H ACTN4 mutation in this patient is not sufficient to confirm with high certainty that this variant is the etiology of this patient’s disease.

## Conclusion

Taken together, we have provided evidence supporting an Y265H ACTN4 mutation as a potential novel FSGS-causing mutation. Our studies demonstrate the need to perform functional assessment of a rare genetic variant before attributing it as causal.
